# First Insights into the Urinary Metabolome of Captive Giraffes by Proton Nuclear Magnetic Resonance Spectroscopy

**DOI:** 10.3390/metabo10040157

**Published:** 2020-04-17

**Authors:** Chenglin Zhu, Sabrina Fasoli, Gloria Isani, Luca Laghi

**Affiliations:** 1 Department of Agro-Food Science and Technology, University of Bologna, 47521 Cesena, Italy; chenglin.zhu2@unibo.it; 2 Department of Veterinary Medical Sciences, University of Bologna, Ozzano Emilia, 40064 Bologna, Italy; sabrina.fasoli2@unibo.it (S.F.); gloria.isani@unibo.it (G.I.)

**Keywords:** captive giraffes, urine, metabolomics, ^1^H-NMR

## Abstract

The urine from 35 giraffes was studied by untargeted ^1^H-NMR, with the purpose of obtaining, for the first time, a fingerprint of its metabolome. The metabolome, as downstream of the transcriptome and proteome, has been considered as the most representative approach to monitor the relationships between animal physiological features and environment. Thirty-nine molecules were unambiguously quantified, able to give information about diet, proteins digestion, energy generation, and gut-microbial co-metabolism. The samples collected allowed study of the effects of age and sex on the giraffe urinary metabolome. In addition, preliminary information about how sampling procedure and pregnancy could affect a giraffe’s urinary metabolome was obtained. Such work could trigger the setting up of methods to non-invasively study the health status of giraffes, which is utterly needed, considering that anesthetic-related complications make their immobilization a very risky practice.

## 1. Introduction

According to the International Union for Conservation of Nature (IUCN), giraffe (*Giraffa camelopardalis*) is declared a vulnerable species [1]. Moreover, different measures have been taken to monitor and protect giraffe population. For example, the International Union for Conservation of Nature (IUCN) Species Survival Commission (SSC) Giraffe and Okapi Specialist Group (GOSG) was established with the aim of studying and guaranteeing the conservation needs of this species (https://www.giraffidsg.org/). In addition, from November 26, 2019, giraffes are included in Appendix II of the CITES (Convention on International Trade in Endangered Species of Wild Fauna and Flora) to improve its protection, subjecting it to strict regulation (https://www.cites.org/).

Zoos represent a significant part of the protection strategy for giraffes, with projects explicitly aimed at protecting endangered species and pursuing high standards of animal welfare [2]. In these structures, however, giraffes may be subjected to sources of stress that reverberate negatively on individual and social behaviors [3]. Causes of stress could be represented by the presence of visitors and attendants [4]. Among the efforts that have been made to reduce the stressors, some are devoted to developing protocols to evaluate their general health status that do not involve immobilization, but are based on indirect methods [3]. In fact, giraffes are particularly prone to anesthetic-related complications and death, due to their unique cardiovascular system, making immobilization a risky practice [5,6].

The possibility of obtaining information from urine collected from the ground seems particularly attractive from this point of view, but the literature on this type of sampling is absent for giraffes and it has been only reported in okapi [7]. Indeed giraffes have been studied more for their iconic height and the mechanisms existing at the cardiovascular level to counterbalance the consequent state of primary hypertension [8,9,10].

Among the completely unexplored characteristics of giraffe urine is its metabolome, the ensemble of low weight molecules produced by the cellular metabolism. Studies carried out by liquid chromatography–mass spectrometry (LC/MS) or by proton nuclear magnetic resonance spectroscopy (^1^H-NMR) on humans and other animals suggest that the giraffe’s urinary metabolome may be particularly informative about the general health of the animal. In horse urine, molecules revealing the action of the intestinal microbiota were found in micromolar concentrations [11,12]. Molecular patterns of the urinary metabolome linked to inflammatory processes have been identified in humans [13]. Urinary profile responses to the calorie content of the diet were identified in rat [14]. The effects of heat stress were studied in cattle by metabolomic profiling of urine [15]. Indeed, the use of urine as a source of biological data in giraffes could be a suitable alternative, due to its non-invasive approach that could avoid the immobilization of animals.

Among the analytical platforms capable of fulfilling the requirements, proton nuclear magnetic resonance spectroscopy (^1^H-NMR) has been widely used for the investigation of urine metabolomes, taking advantage of its high reproducibility and minimal sample preparation.

In the present study, we wanted to verify the feasibility of ^1^H-NMR based metabolomic studies focusing on the urine of giraffes. For this purpose, we characterized the molecular profile of healthy giraffes held in captivity to obtain preliminary quantitative values that could be applied for the diagnosis of diseases affecting this animal. Moreover, the samples collected gave the opportunity to have a first insight about the influence of important physiological factors, such as the sex and age of the subjects, on the urinary metabolomic profile.

## 2. Results

### 2.1. Urinary Metabolites Identification by Untargeted ^1^H-NMR

**Figure 1:**
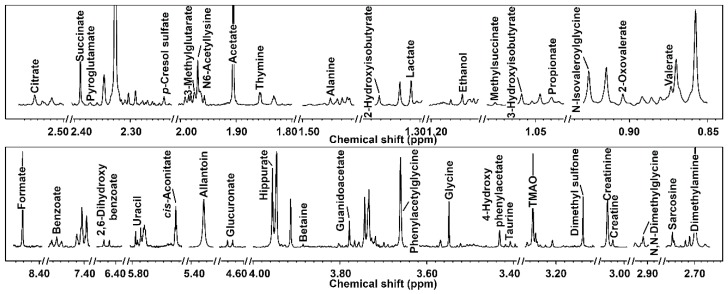
Portions of ^1^H-NMR spectra, representative of all the spectra obtained in this study. Each molecule’s name appears over the NMR peak used for its quantification. To ease the visual inspection of each portion, a different spectrum with a convenient signal-to-noise ratio has been selected.

A representative spectrum of the metabolites identified in the giraffe’s urine is reported in Figure 1. In this study, we identified 39 molecules (Table S1). These molecules mainly pertain to the classes of amino acids and derivatives and organic acids and derivatives. Hippurate (30.63%), creatinine (25.17%), and phenylacetylglycine (12.64%) were the most represented metabolites.

### 2.2. Effects of Sampling Procedure and Location

**Figure 2:**
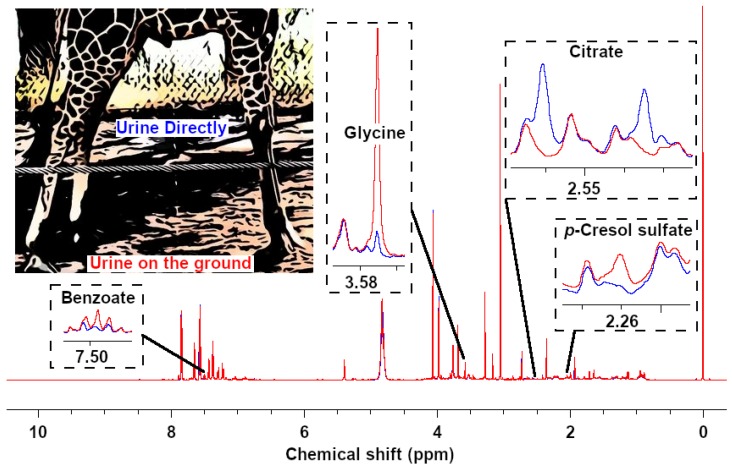
Representative sections of two spectra obtained from analyzing urine from the same giraffe (Ronny), collected directly (**blue line**) and from the ground (**red line**) during one urination, respectively.

To check the potential influence of the different sampling methods, we wanted to collect pairs of samples during the same voiding, one directly and one from the ground. Unfortunately, we only succeeded in this task for one individual (Ronny). Among 39 quantified compounds, four molecules showed a variation of concentration higher than 50%, namely *p*-cresol sulfate, citrate, glycine, and benzoate. ^1^H-NMR signals for these compounds are reported in Figure 2. In detail, benzoate and glycine were more concentrated in the urine collected from the ground, while citrate and *p*-cresol sulfate showed the opposite trend. Overall, the 39 molecules showed a median difference between the two samples of 4.8%. As these observations were based only on one pair of samples from a single individual, we decided not to exclude these molecules from the subsequent analyses. 

To obtain hints about the potential effects of location on the metabolome of giraffe urine, we selected the samples from the locations BG (Parco Faunistico Le Cornelle) and FA (Zoosafari Fasanolandia), where most of the samples had been collected, and we set up a three-way ANOVA analysis aiming at excluding any effect related to gender or age. None of the molecules quantified appeared as significantly different in relation to zoo, so this variable was not considered in the subsequent analyses.

### 2.3. Sex Affects the Giraffe Urine Molecular Profile

To obtain preliminary data on the effect of sex on the urinary metabolome, we focused on samples collected from adult, non-pregnant individuals. Six molecules were found to be significantly (*p* < 0.05) affected by sex, as shown in Table 1. 

**Figure 3:**
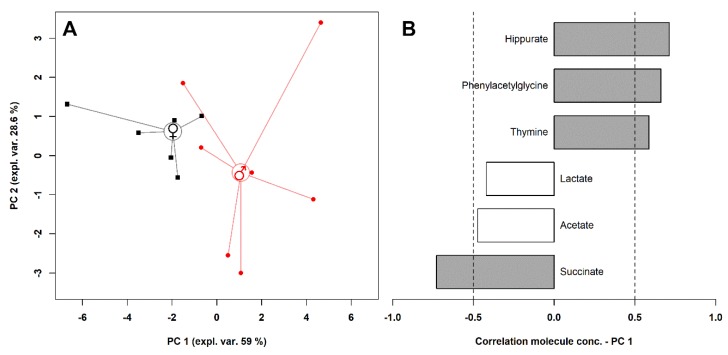
rPCA model calculated on the concentration of the significantly different molecules between male and female giraffes. The scoreplot (**A**) represents with squares and circles females and males, respectively. The median of each sample group is represented by wide circles. The loading plot (**B**) reports the correlation between the importance of each substance over principal component 1 and its concentration. Gray bars highlight significant correlations (*p* < 0.05).

To have an overall view of the data, a robust principal component analysis (rPCA) model was calculated on their concentration, as shown in Figure 3.

Three principal components (PCs) were accepted by the algorithm to depict the overall data features. PC 1, accounting for 59% of the variance thus represented, indeed significantly summarized the peculiarities connected to sex (*p* < 0.05), with female and male individuals appearing respectively at low and high PC scores. Among these molecules, hippurate, phenylacetylglycine, and thymine were more abundant in the urine of male individuals, while lactate, acetate, and succinate were more concentrated in the females’ urine. 

### 2.4. Effect of Age on the Urinary Metabolome

**Figure 4:**
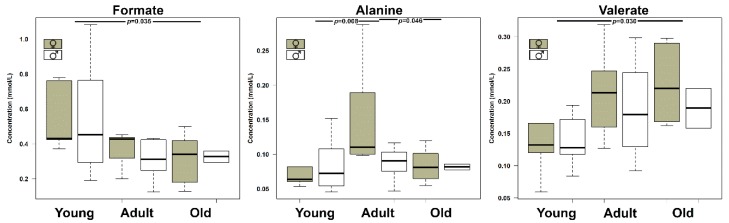
Boxplots showing the concentration of molecules significantly (*p* < 0.05) affected by age, as assessed by two-way ANOVA followed by Tukey post-hoc test.

**Figure 5:**
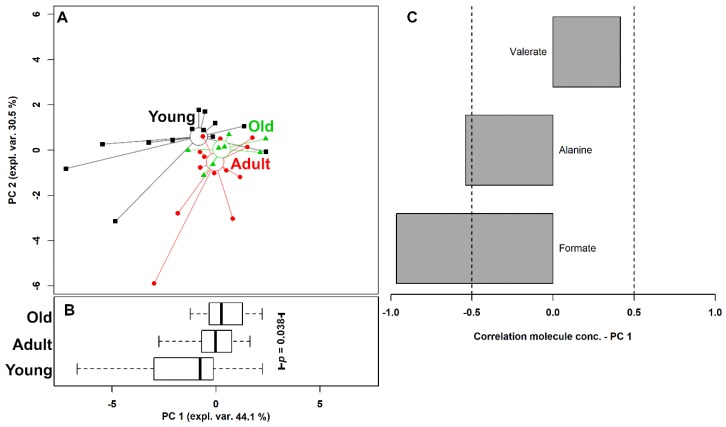
rPCA model of the concentration of the molecules showing a significant difference among the giraffes grouped by age. The scoreplot (**A**) shows the samples from the three groups with squares (Young), circles (Adult), and triangles (Old). The median of each sample group is represented by wide circles. The boxplot (**B**) summarizes the positions of the samples along PC1 and compares them by two-way ANOVA, followed by Tukey post-hoc test. The loading plot (**C**) reports the correlation between the importance of each substance over PC 1 and its concentration. Gray bars highlight significant correlations (*p* < 0.05).

Age was found to significantly affect (*p* < 0.05) the concentration of three urinary metabolites, namely formate, alanine, and valerate, (Figure 4). To understand if their evolution was part of a trend spanning over the entire life of the giraffe, these molecules were used as a base for an rPCA model (Figure 5).

Three PCs were accepted by the algorithm to depict the overall data features. PC 1, accounting for 44.1% of the variance thus represented, summarized effectively the peculiarities connected to age (*p* < 0.05), with Young, Adult, and Old individuals appearing respectively at low, intermediate, and high PC scores. Among these molecules, formate and alanine were more abundant in young individuals, while valerate showed an opposite trend.

### 2.5. Pregnancy Related Urinary Metabolome

Urine samples were obtained from two female giraffes during and after pregnancy (Table S2). Despite the limited number of samples, it was possible to observe a variation of five metabolites during the pregnancy. These molecules showed consistent trends in the samples from both giraffes. All these molecules showed a relevant increase in concentration during the pregnancy, except for phenylacetylglycine, as shown in Table 2.

## 3. Discussion

The present paper describes one of the first studies ever devoted to the urinary metabolome of nonfarmed animals, and the very first focusing on the giraffe metabolome. Due to such paucity of studies on the topic, a key point that needs to be addressed before giraffe urine can be used for metabolomics studies is the possibility of relying on samples collected from the ground. Several aspects, in fact, make the collection of urine directly from the individual during urination highly impractical. To obtain a first insight on this point, we managed to collect the same urine sample either at the start of a spontaneous voiding or from the ground with a syringe at the end the voiding. The corresponding ^1^H-NMR spectra were highly superimposable, except for four molecules, namely benzoate, citrate, *p*-cresol sulfate, and glycine. The fact that the non-volatile glycine showed the greatest differences gave hints that the discrepancies could be mainly connected to dynamic variations in composition during urination, in agreement with Sink and Weinstein [16]. Modifications induced by the collection method could therefore be considered a confounding factor of lower entities than inhomogeneity in the composition of urine during voiding.

The 39 molecules identified give information about protein digestion, diet, gut-microbial co-metabolism, and energy production. Their quantitative observation therefore offers a handy perspective of the health status of giraffes, through a quintessentially non-invasive sampling method.

Comparisons with the urinary metabolome of other animals are also possible, giving indirect information about the differences in metabolism. An example of this possibility is offered by allantoin. This molecule is the fourth most concentrated in giraffe urine (Table S1), identically to yak (*Bos grunniens*) [17] and horse [18]. Differently from these strictly herbivorous animals, this molecule is the most concentrated in the urine of the giant panda [19], even if the giant panda consumes an amount of vegetables in relation to body weight (as much as 30%) much higher than ruminants or horses, which should lead to the lowest concentration of urinary allantoin [20]. This apparent contradiction leads to speculate that the main mechanism determining the concentration of allantoin in the urine of the above-mentioned animals is likely to be its renal reabsorption, which is very effective in strictly herbivorous animals [20].

### 3.1. Sex Affects the Giraffe Urine Molecular Profile

In the current study acetate, succinate, and lactate concentrations appeared to be significantly higher in female giraffe urine, while hippurate, phenylacetylglycine, and thymine were more concentrated in male urine. For acetate, two of the authors of the present paper identified a similar situation in horse urine [18]. For the other molecules, indirect connections with published findings can be devised. There is an abundance of references, focusing on humans, showing that exercise leads to higher concentrations of acetate, succinate, and lactate in urine, and lower concentrations of thymine and hippurate [21,22,23]. Ginnett et al. showed that female giraffes spend more time walking, foraging, feeding, and traveling than males [24]. The two observations seem to suggest that the sex-related differences observed in the urine of males and females may be partly due to the different daily activities. Contrary to the previously reported molecules, phenylacetylglycine is mainly a co-metabolite of gut microorganisms, derived from valine, leucine, phenylalanine, lysine, or ornithine [25]. Its different concentration in relation to sex may therefore reflect peculiarities in gut microbiota profiles or different foraging behaviors, similarly to what was recently observed in the giant panda [19]. Ginnett et al., in fact, demonstrated that males prefer larger bites than females, with potential consequences on the food, and in turn urine, metabolome profile [24]. It is tantalizing to speculate that the length of the neck, which is higher in males [5], may play a role too. In fact, Schüßler and Greven [26] found an allometric direct relationship between rumen-to-mouth distance and the duration of rumination intercycles, influencing in turn the digestive action of ruminal microorganisms.

### 3.2. Effect of Age

By removing the gender effect by two-way ANOVA, it was possible to focus on the effect of age. In parallel with previous studies in rats and humans [27,28], formate and alanine were negatively related to age. The trend observed for formate is very likely related to the gut microbiome. In fact, in the gut microbiota of the juvenile giraffes there is a prevalence of *Bacteroides* and *Acinetobacter* genera, responsible for the degradation of starch and cellulose to formate [29], while in the gut of adult giraffes other genera tend to prevail, such as *Treponema* [30].

The concentration of amino acids in urine has been consistently linked to the turnover of muscle amino acids [18,31], with urinary concentration of alanine specifically related to exercise [32]. Therefore, the difference in the concentration of alanine could be ascribed to a variation of daily activity intensity along age.

### 3.3. Effect of Pregnancy

Early identification of pregnant giraffes with maximum accuracy is an important issue for optimizing their management. Although some diagnostic methods (e.g., ultrasonography) have been described in domestic animals [33], their application to wild or captive animals is hindered by practical reasons. Metabolomics approaches seem in principle promising for setting up diagnostic methods that might be more convenient in specific contexts, due to the possibility to quantify a high number of molecules at the same time. However, previous studies performed in domestic animals were focused on serum [34,35], a sub-optimal sample from the point of view of non-invasivity. Therefore, despite the restricted number of samples analyzed in the present study, the obtained data can provide a preliminary urinary fingerprint of pregnancy in giraffes. 

Taurine is an important amino acid during pregnancy and lactation, because it satisfies the needs of both the fetus and suckling infant. In our research, taurine excretion through urine increased during early pregnancy, consistent with human studies [36]. Taurine is rarely found in plants [37], so that herbivores cannot obtain a sufficient amount taurine from the diet. Remarkably, in ruminants the urinary taurine concentration is strongly diet-dependent, as can be inferred from the works of Bristow et al. on cows fed with maize silage compared to free grazing cows [38]. Diet is therefore likely to trigger biosynthetic pathways, such as the one leading to taurine from methionine [39]. Moreover, a specific pathway, converting homocysteine to taurine and glycine through cysteine, is known to become effective in early pregnancy [39]. This latter mechanism is a likely reason for the increasing trend of taurine excretion we found in the present work.

A further contribution to urine metabolome profile modifications may be due to changes in the gut microbiota. In fact, among the molecules showing the greatest changes we found *p*-cresol sulfate and phenylacetylglycine, mainly described as gut microorganism co-metabolites [11,25], absorbed at the intestinal level and then expelled through urine. Interestingly, the change in the concentration of both has been related, in humans, with alterations in the microbiota profile linked to inflammatory states [13,40], in which pregnancy is known to play a role [41]. Despite the very limited number of cases here, these observations support the compelling possibility to use the urine metabolome to gain specific information about giraffe inflammatory status during pregnancy, as modulated by the gut microbiota.

## 4. Materials and Methods 

### 4.1. Compliance with Ethical Requirements

All the procedures related to animals respected the Directive 2010/63/EU of the European Parliament and of the Council of September 22, 2010 on the protection of animals used for scientific purposes (Article 1, Paragraph 1, Letter b) and the Italian legislation (D. Lgs. n. 26/2014, Article 2, Paragraph 1, Letter b).

### 4.2. Sample Collection

A total of 35 captive giraffes (*Giraffa camelopardalis*) were involved in the current study. Based on physical examinations, giraffes did not show symptoms of diseases both before and during the urine sampling period. The giraffes were housed in five Italian zoos: Zoosafari Fasanolandia (FA) (N = 11), Safari Ravenna (RA) (N = 4), Giardino Zoologico di Pistoia (PT) (N = 1), Parco Natura Viva (VR) (N = 4), and Parco Faunistico Le Cornelle (BG) (N = 15).

The details for each giraffe are reported in Table 3. Their age ranged from a minimum of 6 months to a maximum of 20 years. The giraffes were categorized in 3 age classes: Young (from 6 months to 6 years old, N = 14), Adult (from 6 to 15 years old, N = 16), and Old (older than 15, N = 9), according to the following information. In female giraffes the first birth is at about 6.4 years old, even if sexual maturity is reached at 3–4 years [42,43]. Giraffe males are considered as adults when older than 6 years old, according to Lee et al. [44]. 

The samples were collected between 10:00 a.m. and 2:00 p.m., in connection to the daily activities of the keepers. Urine samples were collected with a syringe from the ground. To limit the soil contaminants, only the upper part of the urine was collected, immediately after the spontaneous voiding, before it was absorbed by the soil. A sample from one male was also collected directly into a sterile beaker, preventing the sample from touching the ground. Four urine samples were collected from two females during and after pregnancy. After collection, the urine samples were centrifuged at 1500× *g* for 10 min, to further remove potential ground contaminants, and the supernatants were frozen at −80 °C. 

### 4.3. Metabolomic Analysis

We prepared urine samples for NMR by thawing and centrifuging them for 15 min at 18,630× *g* at 4 °C. We added the supernatant (350 μL) to bi-distilled water (350 μL) and to a D_2_O solution (200 μL) of TSP (3-(trimethylsilyl)-propionic-2,2,3,3-d4 acid) 10 mM and of NaN_3_ 2 mM. A 1M phosphate buffer had been used to set the D_2_O solution to pH of 7.00 ± 0.02. After a further centrifugation, we recorded ^1^H-NMR spectra at 298 K with an AVANCE III spectrometer (Bruker, Milan, Italy), at a frequency of 600.13 MHz, equipped with Topspin software (Ver. 3.5).

According to Zhu et al. [17], we suppressed the signals from broad resonances using a CPMG-(Carr-Purcell-Meiboom-Gill) filter composed of 400 echoes with a of 400 s and a 180° pulse of 24 s, for a total filter of 330 ms. We also applied pre-saturation, to reduce the signal from water. We employed Topspin software to apply a line broadening of 0.3 Hz and to adjust the phase of each spectrum. We set the recycle delay to 5 s, by considering the relaxation time of the protons under investigation. We employed R computational language [45] for any further processing of spectra, quantification of molecules, and data mining, with custom scripts.

We aligned the spectra by using the TSP signal as a reference (−0.017 ppm). We adjusted the baseline of each spectrum by distinguishing irregularities of the baseline from genuine signals, according to the “rolling ball” idea [46], implemented in the R package “baseline” [47]. We performed the assignment of the signals by comparing chemical shift and multiplicity with the libraries (Ver. 10) of Chenomx software (Chenomx Inc., Canada, v. 8.3). 

According to Dieterle et al. [48], water intake behavior can change the dilution of urine as much as five times, obscuring any trend in metabolite concentrations. We removed this confounding factor by calculating, for each sample, the ratio between the area of TSP peak and the intensity of the spectrum. This allowed us to estimate the dilution of each sample and to select the one with the mostly representative dilution. We used this sample as a reference by quantifying the molecules from the added TSP. We then normalized the other samples towards the reference by probabilistic quotient normalization (PQN) [48].

### 4.4. Statistical Analysis

We conducted the statistical analysis in R computational language [45] and we refined the artwork by GIMP (version 2.10, www.gimp.org). Prior to univariate analysis, we transformed the data to normality by BoxCox transformation [49]. To investigate the effects of sex on urinary metabolites, we considered only adult, non-pregnant giraffes. This allowed us to reduce potential interferences due to different age classes. We then highlighted any difference by t-test. To investigate age related effects, by removing sex effect, we applied a two-way ANOVA test followed by Tukey-HSD, by taking advantage of the “aov” function of the R package “stats” [50]. For the above statistical tests, we accepted a cut-off *p*-value of 0.05. 

In agreement with Bazzano et al. [51], we highlighted any trend characterizing the samples with robust principal component analysis (rPCA) models [52], using the molecules accepted by univariate analysis as a base. We took advantage of the PcaHubert algorithm implemented in the “rrcov” package. The algorithm grants robustness with a two-steps approach. In the first step outlying samples are detected according to their distance from the others along and orthogonally to the PCA plane. A second step determines the optimal number of principal components (PCs). The main features of each rPCA model are summarized by a scoreplot and by a Pearson correlation plot. The former is the projection of the samples in the PC space and highlights the underlying structure of the data. The latter relates the concentration of each variable to the components of the model.

## 5. Conclusions

This work represents a primer in giving quantitative information about the urinary metabolome of captive giraffes, as detected by untargeted ^1^H-NMR. Foraging behaviors and daily activity could be considered as one of the main reasons for the differences we highlighted that are linked to sex and age. A preliminary observation conducted on two female giraffes suggests that ^1^H-NMR based metabolomics could be conveniently applied to monitor modifications occurring during pregnancy, some of which are potentially related to inflammatory status triggered by modification of the microbiota profile. 

## Figures and Tables

**Table 1:** Metabolite concentrations (mmol/L, median (IQR)) in the adult group were significantly (*p* < 0.05) affected by sex, as assessed by *t*-test.

	Females (6)	Males (7)	Trend	*p* Value
Acetate	2.04 (5.23 × 10^−1^)	1.33 (9.04 × 10^−1^)	↓	0.034
Hippurate	13.50 (10.70)	19.30 (19.50)	↑	0.047
Lactate	2.77 × 10^−1^ (8.90 × 10^−2^)	1.28 × 10^−1^ (7.35 × 10^−2^)	↓	0.003
Phenylacetylglycine	7.82 (2.41)	15.20 (5.53)	↑	0.014
Succinate	2.48 × 10^−1^ (3.00 × 10^−2^)	1.66 × 10^−1^ (8.80 × 10^−2^)	↓	0.006
Thymine	1.77 × 10^−1^ (4.94 × 10^−2^)	2.86 × 10^−1^ (1.79 × 10^−1^)	↑	0.043

**Table 2:** Urinary metabolites (mmol/L) affected by pregnancy consistently across the two giraffes observed.

	Giulietta		Nicole	
	Not Pregnant	Pregnant ^1^	Not Pregnant	Pregnant
Phenylacetylglycine	10.20	3.52 ↓	10.40	5.02 ↓
Benzoate	2.14	3.88 ↑	2.46	12.22 ↑
Glycine	1.06	3.04 ↑	1.79	11.65 ↑
Taurine	1.75 × 10^−1^	2.93 × 10^−1^ ↑	7.98 × 10^−2^	1.33 × 10^−1^ ↑
*p*-Cresol sulfate	1.46 × 10^−2^	2.15 × 10^−2^ ↑	6.37 × 10^−2^	3.50 × 10^−1^ ↑

^1^ For readability, only molecules changing by more than 40% for both giraffes are shown.

**Table 3:** Animal information.

Sample ID	Name	Sex	Age (years)	Zoo
N.01	Ronny	Male	14	FA
N.02	Nicole	Female	14	FA
N.03	Giulietta	Female	17	FA
N.04	Marcello	Male	9	FA
N.05	Italia	Female	8	FA
N.06	Carlos	Male	2	RA
N.07	Daniele	Male	11	RA
N.08	Cleopatra	Female	20	PT
N.09	Alto	Male	2	FA
N.10	Congo	Male	0.3	FA
N.11	Roberto	Male	0.6	RA
N.12	Martina	Female	0.6	RA
N.13	Linda	Female	16	BG
N.14	Sandy	Female	16	BG
N.15	Raffa	Female	7	BG
N.16	Telete	Female	2	BG
N.17	Rusman	Male	16	BG
N.18	Akuna	Female	10	BG
N.19	Ciokwe	Male	5	BG
N.20	Miro	Male	9	BG
N.21	Lucia	Female	16	BG
N.22	Nuvola	Female	7	BG
N.23	Sahel	Female	2	BG
N.24	Russel	Male	16	BG
N.25	Ramiro	Male	3	BG
N.26	Madiba	Male	6	BG
N.27	Nasanta	Female	2	BG
N.28	Macchia	Male	5	VR
N.29	Secondo	Male	11	VR
N.30	Akasha	Male	7	VR
N.31	Quarto	Male	9	VR
N.32	Luna	Female	15	FA
N.33	Kenya	Female	20	FA
N.34	Alessia	Female	4	FA
N.35	Mina	Female	14	FA

## Supplementary Materials

The following are available online at https://www.mdpi.com/2218-1989/10/4/157/s1, Table S1: Concentration (mmol/L, median (IQR)) of the molecules quantified by ^1^H-NMR in all the samples studied in the present investigation, sorted by abundance. Table S2. Concentration (mmol/L) of the molecules quantified by ^1^H-NMR in the samples collected during and after pregnancy.
