# Supplementary material for: First Insights into the Urinary Metabolome of Captive Giraffes by Proton Nuclear Magnetic Resonance Spectroscopy
Source: Metabolites. 2020 Apr 17;10(4):157. doi: 10.3390/metabo10040157 (PMC7240958; doi:10.3390/metabo10040157)
Supplement: Supplementary file 1 [file metabolites-10-00157-s001.pdf]

First insights into the urinary metabolome of captive giraffe by proton nuclear magnetic resonance spectroscopy

Chenglin Zhu[1#], Sabrina Fasoli[2#], Glori Isani[2], Luca Laghi[1*]

[1]Department of Agro-Food Science and Technology, University of Bologna, 47521, Cesena, Italy

[2]Department of Veterinary Medical Sciences, University of Bologna, Ozzano Emilia, 40064 Bologna, Italy

Table S1. Concentration (mmol/L, median (IQR)) of the molecules quantified by ^{1}H-NMR in all the samples studied in the present investigation, sorted by abundance.

Molecule	Concentration
Creatinine	20.9 (2.00)
Hippurate	19.1 (5.81)
Phenylacetylglycine	8.95 (2.13)
Allantoin	6.87 (2.46)
Benzoate	2.13 (7.38x10^{-1})
Acetate	1.49 (7.27 x10^{-1})
Glycine	1.15 (6.96 x10^{-1})
Glucuronate	8.68x10^{-1} (2.10x10^{-1})
Trimethylamine N-oxide	7.90x10^{-1} (6.22x10^{-1})
Dimethyl sulfone	7.40x10^{-1} (3.11x10^{-1})
Guanidoacetate	7.24x10^{-1} (1.59x10^{-1})
4-Hydroxyphenylacetate	5.84x10^{-1} (2.89x10^{-1})
Dimethylamine	5.14x10^{-1} (3.10x10^{-1})
N6-Acetyllysine	4.95x10^{-1} (1.20x10^{-1})
3-Methylglutarate	4.24x10^{-1} (1.08x10^{-1})
Formate	4.00x10^{-1} (1.56x10^{-1})
N-Isovaleroylglycine	2.90x10^{-1} (5.40x10^{-2})
cis-Aconitate	2.77x10^{-1} (5.18x10^{-2})
Pyroglutamate	2.62x10^{-1} (8.77x10^{-2})
Propionate	2.58x10^{-1} (1.38x10^{-1})
Taurine	2.02x10^{-1} (1.93x10^{-1})
Thymine	1.81x10^{-1} (7.96x10^{-2})
Lactate	1.75x10^{-1} (1.16x10^{-1})
Valerate	1.66x10^{-1} (6.32x10^{-2})
Betaine	1.64x10^{-1} (3.87x10^{-2})
3-Hydroxyisobutyrate	1.50x10^{-1} (1.93x10^{-2})

Succinate	$1.49x10^{-1}$ ($7.69x10^{-2}$)
Creatine	$1.45x10^{-1}$ ($3.64x10^{-2}$)
Ethanol	$1.17x10^{-1}$ ($4.65x10^{-2}$)
2-Oxovalerate	$1.14x10^{-1}$ ($2.84x10^{-2}$)
Sarcosine	$1.08x10^{-1}$ ($4.99x10^{-2}$)
2,6-Dihydroxybenzoate	$1.02x10^{-1}$ ($2.65x10^{-2}$)
Alanine	$8.55x10^{-2}$ ($2.68x10^{-2}$)
Citrate	$6.56x10^{-2}$ ($2.14x10^{-2}$)
N,N-Dimethylglycine	$6.27x10^{-2}$ ($6.07x10^{-2}$)
2-Hydroxyisobutyrate	$5.81x10^{-2}$ ($4.12x10^{-2}$)
Uracil	$5.55x10^{-2}$ ($1.83x10^{-2}$)
p-Cresol sulfate	$4.86x10^{-2}$ ($4.10x10^{-2}$)
Methylsuccinate	$2.43x10^{-2}$ ($7.11x10^{-3}$)

Table S2. Concentration (mmol/L) of the molecules quantified by ^{1}H-NMR in the samples collected during and after pregnancy.

	Giulietta		Nicole	
	**Not Pregnant**	**Pregnant**	**Not Pregnant**	**Pregnant**
Formate	$2.91x10^{-1}$	$3.03x10^{-1}$	$2.00x10^{-1}$	$2.82x10^{-1}$
Benzoate	2.14	3.88	2.46	12.22
2,6-Dihydroxybenzoate	$9.83x10^{-2}$	$1.82x10^{-1}$	$1.10x10^{-1}$	$1.07x10^{-1}$
Uracil	$6.27x10^{-2}$	$8.18x10^{-2}$	$8.60x10^{-2}$	$5.76x10^{-2}$
cis-Aconitate	$2.72x10^{-1}$	$2.55x10^{-1}$	$2.19x10^{-1}$	$2.09x10^{-1}$
Allantoin	6.76	9.86	9.87	6.53
Glucuronate	$7.18x10^{-1}$	$4.70x10^{-1}$	$3.70x10^{-1}$	$3.64x10^{-1}$
Hippurate	24.7	21.6	23.00	8.11
Betaine	$2.01x10^{-1}$	$1.46x10^{-1}$	$1.29x10^{-1}$	$1.24x10^{-1}$
Guanidoacetate	$8.07x10^{-1}$	$7.54x10^{-1}$	$4.95x10^{-1}$	$7.05x10^{-1}$
Phenylacetylglycine	10.20	3.52	10.40	5.02
Glycine	1.06	3.04	1.79	11.65
Acetoacetate	$4.27x10^{-2}$	$3.70x10^{-2}$	$4.33x10^{-3}$	$8.08x10^{-3}$
4-Hydroxyphenylacetate	$3.55x10^{-1}$	$3.62x10^{-1}$	$6.19x10^{-1}$	$7.81x10^{-1}$
Taurine	$1.75x10^{-1}$	$2.93x10^{-1}$	$7.98x10^{-2}$	$1.33x10^{-1}$
Trimethylamine N-oxide	1.36	1.38	1.52	1.44
Dimethyl sulfone	$9.76x10^{-1}$	1.09	$4.64x10^{-1}$	1.17
Creatinine	13.1	15.80	12.90	14.10
Creatine	$1.17x10^{-1}$	$9.12x10^{-2}$	$1.27x10^{-1}$	$1.22x10^{-1}$
N,N-Dimethylglycine	$6.22x10^{-2}$	$9.52x10^{-2}$	$2.49x10^{-2}$	$1.28x10^{-1}$
Sarcosine	$1.74x10^{-1}$	$1.66x10^{-1}$	$1.12x10^{-1}$	$2.01x10^{-1}$
Dimethylamine	$5.16x10^{-1}$	$9.48x10^{-1}$	$8.00x10^{-1}$	$9.72x10^{-1}$
Citrate	$3.14x10^{-2}$	$2.70x10^{-2}$	$3.82x10^{-2}$	$4.07x10^{-2}$
Succinate	$1.32x10^{-1}$	$5.26x10^{-2}$	$1.98x10^{-1}$	$1.56x10^{-1}$

Pyroglutamate	$3.28 x 10^{-1}$	$3.24 x 10^{-1}$	$2.64 x 10^{-1}$	$2.29 x 10^{-1}$
p-Cresol	$1.46 x 10^{-2}$	$2.15 x 10^{-2}$	$6.37 x 10^{-2}$	$3.50 x 10^{-1}$
3-Methylglutarate	$4.59 x 10^{-1}$	$4.66 x 10^{-1}$	$4.93 x 10^{-1}$	$4.12 x 10^{-1}$
N6-Acetyllysine	$5.71 x 10^{-1}$	$5.95 x 10^{-1}$	$5.46 x 10^{-1}$	$4.70 x 10^{-1}$
Acetate	1.94	1.47	1.57	3.97
Thymine	$1.73 x 10^{-1}$	$1.36 x 10^{-1}$	$1.58 x 10^{-1}$	$1.40 x 10^{-1}$
Alanine	$6.47 x 10^{-2}$	$6.65 x 10^{-2}$	$1.86 x 10^{-1}$	$1.85 x 10^{-1}$
2-Hydroxyisobutyrate	$1.04 x 10^{-1}$	$4.72 x 10^{-2}$	$3.23 x 10^{-2}$	$3.25 x 10^{-2}$
Lactate	$6.83 x 10^{-2}$	$1.52 x 10^{-1}$	$2.12 x 10^{-1}$	$1.46 x 10^{-1}$
Ethanol	$1.17 x 10^{-1}$	$1.09 x 10^{-1}$	$1.15 x 10^{-1}$	$1.41 x 10^{-1}$
Methylsuccinate	$1.34 x 10^{-2}$	$1.62 x 10^{-2}$	$1.58 x 10^{-2}$	$2.24 x 10^{-2}$
3-Hydroxyisobutyrate	$1.57 x 10^{-1}$	$1.52 x 10^{-1}$	$1.64 x 10^{-1}$	$1.42 x 10^{-1}$
Propionate	$3.04 x 10^{-1}$	$1.71 x 10^{-1}$	$1.48 x 10^{-1}$	$6.24 x 10^{-1}$
N-Isovaleroylglycine	$2.92 x 10^{-1}$	$2.68 x 10^{-1}$	$3.12 x 10^{-1}$	$2.77 x 10^{-1}$
2-Oxovalerate	$9.38 x 10^{-2}$	$1.35 x 10^{-1}$	$9.90 x 10^{-2}$	$1.25 x 10^{-1}$
Valerate	$1.68 x 10^{-1}$	$1.63 x 10^{-1}$	$2.45 x 10^{-1}$	$1.98 x 10^{-1}$
